# Association Between Allergic Rhinitis and Children With Appendectomy—A Nationwide Population‐Based Retrospective Cohort Study

**DOI:** 10.1002/hsr2.71073

**Published:** 2025-07-18

**Authors:** Wen‐Chun Lin, Meng‐Che Wu, Yu‐Hsun Wang, James Cheng‐Chung Wei

**Affiliations:** ^1^ Institute of Medicine Chung Shan Medical University Taichung Taiwan; ^2^ Division of Otolaryngology Head and Neck Surgery Taichung Veterans General Hospital Taichung Taiwan; ^3^ Department of Post‐Baccalaureate Medicine, College of Medicine National Chung Hsing University Taichung Taiwan; ^4^ Division of Pediatric Gastroenterology, Children's Medical Center Taichung Veterans General Hospital Taichung Taiwan; ^5^ School of Medicine Chung Shan Medical University Taichung Taiwan; ^6^ Department of Medical Research Chung Shan Medical University Hospital Taichung Taiwan; ^7^ Department of Allergy, Immunology and Rheumatology Chung Shan Medical University Hospital Taichung Taiwan; ^8^ Graduate Institute of Integrated Medicine China Medical University Taichung Taiwan

**Keywords:** allergic rhinitis, appendectomy, cohort study, National Health Insurance Research Database

## Abstract

**Background and Aims:**

The appendix plays an important role to the human intestinal microbiota and immunity. Thus, appendectomy may alter immune function and intestinal biofilm. Allergic rhinitis is a common disease which is due to imbalance of TH2/TH1 cells and cytokine. The aim of this study was to evaluate the risk of allergic rhinitis after appendectomy in children.

**Methods:**

The data source was the National Health Insurance Research Database (NHIRD), which is a large, population‐based database in Taiwan. We collected 4013 patients who had undergone appendectomy (case group) between January 1, 2000 and December 31, 2018 and matched them with 16,052 patients who had not undergone appendectomy (control group) by sex and age using proportional propensity score (PSM) at a ratio of 1:4 after excluding patients with a diagnosis of allergic rhinitis in the year prior to appendectomy. In addition, Poisson regression and subgroup analyses were used to investigate the relative risk of the development of allergic rhinitis after appendectomy in children.

**Results:**

The relative risk of subsequent allergic rhinitis in patients who had undergone appendectomy was found to be higher (RR = 1.24; *p* < 0.001) than in patients who had never undergone appendectomy. Subgroup analysis showed the risk of allergic rhinitis after appendectomy was significantly higher in patients aged 6–11 years and 12–18 years, and for both genders, living place, and lower income groups. When stratified by follow‐up duration, the risk of developing allergic rhinitis within 5 years of follow‐up was also significantly higher in the exposed group than in the control group.

**Conclusion:**

Appendectomy was correlated with a 1.24‐fold increased risk of developing allergic rhinitis in children, especially within 5 years of follow‐up. Therefore, we suggest that the indication of appendectomy should be carefully evaluated to decide the best way to treat acute appendicitis.

## Summary

- Children who underwent appendectomy are at higher risk of developing allergic rhinitis than children without appendectomy, especially within 5 years after surgery.
- Therefore, the necessity of appendectomy should be carefully evaluated in children.
- Patients should be closely monitored for symptoms of allergic rhinitis after appendectomy and treated in time.

## 1 Introduction

In the past, the human appendix was considered a useless remnant structure of the intestinal tract, and thus in the treatment of appendicitis, appendectomy was considered an easy and straightforward decision [1, 2, 3]. Appendix vermiformis provides an appropriate environment for microbiota growth, prevents the adhesion of other potentially pathogenic microorganisms to the gut's epithelium, and may restore intestinal microbial diversity and stability after a gastrointestinal infection or after a period of antibiotic use [2, 4, 5, 6, 7, 8]. Normal appendix plexus is composed mainly of Firmicutes, Bacteroidetes, Actinobacteria, and Proteus which is similar to the populations seen in the colon [2, 7]. For this reason, the appendix is known as a “protective umbrella” for normal intestinal microbiota. Gut‐associated lymphoid tissue (GALT) is abundant over the tonsils and Peyer's patches, and is also highly concentrated in the appendix [2, 3, 4, 8]. Many of the B and T lymphocytes shifted from the appendix to the lamina propria of the gut, and B cells mainly differentiate by becoming IgA‐secreting cells in the appendix [2, 9]. Therefore, it is also known as the “abdominal tonsil” [1, 3, 10]. Appendectomy may also alter immune function, and there have been numerous studies trying to the association between appendectomy and autoimmune diseases such as systemic lupus erythematosus, rheumatoid arthritis, inflammatory bowel disease, sepsis, and cancer [4, 5, 6, 7, 9, 10].

Allergic rhinitis affects 30%–40% of the population worldwide and is a common health problem [11, 12, 13, 14, 15, 16]. It results in shortened work hours and sleep disturbance in adults around the world. In children, it reduces school time and outdoor activities [12, 17]. The economic impact of allergic rhinitis is often underestimated because of the high indirect costs it causes [13]. Allergic rhinitis is characterized by sneezing, nasal discharge, nasal congestion, and nasal itching. It is caused by immunoglobulin E (IgE)‐induced reactions to inhalation of allergens. These immune responses involved mucosal inflammation and are primarily driven by type 2 T‐helper (TH2) cells. Dendritic cells in the nasal mucosa come into contact with the allergen when a patient is first exposed to an allergen. It is processed and transported to the lymph nodes, where it is presented to naive CD4+ T lymphocytes. These are activated and differentiated into allergen‐specific TH2 cells. TH2 cells induce B lymphocytes to differentiate into plasma cells that produce allergen‐specific IgE. IgE binds to high‐affinity IgE receptors (FcεRI) on the surface of mast cells and basophils. These sequential processes lead to the formation of allergen‐specific memory TH2 cells and B lymphocytes, resulting in the various symptoms of allergic rhinitis following reexposure to allergens [12, 13, 15, 16, 17, 18]. Asthma and allergic rhinitis shared the common chronic immunopathological pathways, similar allergens and continuous airway mucosa. Allergic rhinitis is recognized as one of the risk factors for asthma in children [15, 18]. Previous studies have suggested that individuals with allergic rhinitis are susceptible to early‐onset severe asthma exacerbations, characterized by poor symptoms control, increased hospitalizations, and frequent emergency room visits [15, 18, 19].

The diverse gut microbiota has an important role in the development of host immunity. Normal gut microbiota disruption or dysregulation leads to the development of immune diseases, such as allergic diseases [11, 12, 13, 16]. A previous study has shown that a relatively lower microbiota diversity exists in patients with allergic rhinitis [12, 16]. Thus, the host–microbe symbiosis plays an important role in maintaining the host's health and immunity [11, 13, 16].

Since the appendix is so important to human intestinal microbiota and immunity, appendectomy may alter immune function and intestinal biofilm. Dysbiosis of the intestinal flora and immune function may also lead to the development of allergic rhinitis. Therefore, we hypothesized that children who received appendectomy would be at higher risk of developing allergic rhinitis in the future. We conducted a nationwide population‐based retrospective cohort study, in which we analyzed data from Taiwan's National Health Insurance Research Database (NHIRD) to test our hypothesis.

## 2 Materials and Methods

The NHIRD in Taiwan was established in 1995. The National Health Insurance (NHI) program is a single‐payer system that covers a wide range of medical costs for more than 99% of Taiwan's population of 23 million. The NHIRD consists of comprehensive de‐identified electronic medical records from the NHI. The database includes general demographics, outpatient and inpatient medical records, details of medication prescriptions, and medical services. Diagnostic and procedure codes were defined according to the International Classification of Diseases, 9th and 10th Revisions, Clinical Modification (ICD‐9‐CM, ICD‐10‐CM) codes. The Research Ethics Committee at Chung Shan Medical University and Hospital in Taiwan approved the study (CSMUH No: CS1‐20201). The consent written from study subjects was proved to be waived by the Institutional Review Board of the Research Ethics Committee of Chung Shan Medical University and Hospital. The study followed the principles of the Declaration of Helsinki.

### 2.1 Study Population

According to literatures, the incidence of acute appendicitis and appendectomy is higher among young people under the age of 30. The incidence increases with age, especially in late teens, when it reaches its peak [20, 21]. To evaluate the association between appendectomy (health insurance procedure codes: 74004B, 97201K, 97202A, 97203B, 74002B) and allergic rhinitis in children (ICD‐9‐CM: 477; ICD‐10‐CM: J30), we used 2,000,000 de‐identified participants in the LHID which is a randomly selected subset of the NHIRD. There were 4829 patients younger than 18 years of age who underwent appendectomy in the period from January 1, 2001 to December 31, 2017. In total, 1,956,074 patients did not have a health insurance procedure code for appendectomy since January 1, 2000 to December 31, 2018. Patients who had a medical record of allergic rhinitis prior to appendectomy were excluded. To ensure the accuracy of diagnosis of allergic rhinitis after appendectomy, only patients diagnosed with allergic rhinitis at least twice as an outpatient or at least once as an inpatient after the index date (i.e., the date of appendectomy and the start date of the follow‐up period) were included.

Covariates for comorbidities included ADHD, autism, asthma, atopic dermatitis, diabetes, congenital heart anomaly, chronic renal diseases, congenital gastrointestinal anomaly disease, chronic liver diseases, constipation, and epilepsy. Furthermore, patients diagnosed with any comorbidity more than once in an outpatient setting or at least once in an inpatient setting prior to the index date were included in the study.

### 2.2 Statistical Analysis

A total of 4829 patients who had undergone appendectomy (case group) were matched with 1,956,074 patients who had not undergone appendectomy (control group) by sex and age using proportional propensity score (PSM) at a ratio of 1:4 after excluding patients with a diagnosis of allergic rhinitis in the year prior to appendectomy. The end point of follow‐up was the date the patient developed allergic rhinitis, expired, withdrew from NHIRD, or December 31, 2018.

Fisher's exact test and Student's *T*‐test were used for the analysis of general demographic and continuous data between groups, with the *t*‐test applied to continuous variables that were normally distributed. Categorical variables were presented as numbers and percentages [*n* (%)], and continuous variables were presented as means and standard deviation (SD). We used Poisson regression to evaluate the relative risk of allergic rhinitis between the two groups. Subgroup analyses were also conducted stratified by sex, age group, urbanization, family income, comorbidities, and follow‐up duration to assess the association between appendectomy and the subsequent risk of allergic rhinitis.

All statistical analyses were performed using SAS software version 9.4 (SAS Institute Inc., Cary, NC), and cumulative incidence curves were generated using R software. The Kaplan–Meier method was used to estimate the cumulative incidence of allergic rhinitis, and the log‐rank test was applied to compare time‐to‐event distributions between the two groups. Univariable and multivariable Cox proportional hazards models were used to calculate hazard ratios (HRs) and 95% confidence intervals (CIs) for the risk of allergic rhinitis between groups, including subgroup and follow‐up period analyses. All statistical tests were two‐tailed, and a *p* < 0.05 was considered statistically significant.

## 3 Results

**Figure 1:**
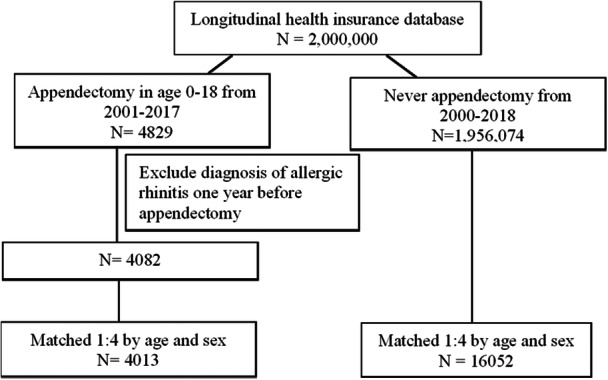
Flowchart for patients with appendectomy and comparison cohort.

For the period January 1st 2000 to December 31st 2018, we collected 4013 patients who had undergone appendectomy and 16,052 who had never undergone appendectomy from the NHIRD in Taiwan. Figure 1 shows the flowchart for patients with appendectomy and comparison cohort. Table 1 shows the demographic characteristics of these patients. Among the two groups, 3%, 25.9%, and 71% of the patients were in the 0–5, 6–11, and 12–18 age subgroups. Approximately 41.6% of patients were female and 58.4% were male. The mean age of both cohorts was 13.5 ± 3.73 years. As for the comorbidities, there were broadly no statistically significant differences between the two cohorts, except for constipation and infectious bowel disease.

**Table 1:** Demographic characteristics of appendectomy and nonappendectomy.

	Nonappendectomy (*N* = 16,052)	Appendectomy (*N* = 4013)	*p* value
	*n* (%)	*n* (%)	*p* value
Age (years)			1
0–5	488 (3)	122 (3)	
6–11	4160 (25.9)	1040 (25.9)	
12–18	11,404 (71)	2851 (71)	
Mean ± SD			1
Sex			1
Female	6680 (41.6)	1670 (41.6)	
Male	9372 (58.4)	2343 (58.4)	
Urbanization			0.01
Urban	9012 (56.1)	2198 (54.8)	
Suburban	5419 (33.8)	1350 (33.6)	
Rural	1621 (10.1)		
Monthly income (family)			0.8
< NT $20,000	8077 (50.3)	2024 (50.4)	
NT $20,001–$39,999	5537 (34.5)	1367 (34.1)	
≥ NT $40,000	2438 (15.2)	622 (15.5)	
ADHD	69 (0.4)	23 (0.6)	0.23
Autism	14 (0.1)	6 (0.1)	0.26
Asthma	43 (0.3)	17 (0.4)	0.1
Atopic dermatitis	32 (0.2)	12 (0.3)	0.22
Diabetes	NA	NA	1
Chronic renal diseases	12 (0.1)	4 (0.1)	0.54
Chronic liver diseases	22 (0.1)	15 (0.4)	0.002
Constipation	54 (0.3)	39 (1)	< 0.001
Intestinal (GI) infectious diseases	102 (0.6)	53 (1.3)	< 0.001
Epilepsy	45 (0.3)	9 (0.2)	0.54

Table 2 shows that the relative risk of subsequent allergic rhinitis in patients who had undergone appendectomy was higher (RR = 1.24; 95% CI, 1.18–1.30; *p* < 0.001) than in patients who had never undergone appendectomy according to the results of Poisson regression analysis.

**Table 2:** Poisson regression of the relative risk of appendectomy and nonappendectomy.

	Nonappendectomy	Appendectomy
*N*	16,052	4013
Person‐years	141,363	33,037
No. of allergic rhinitis	6934	2004
ID (95% CI)	49.05 (47.91–50.22)	60.66 (58.06–63.37)
Relative risk (95% CI)	Reference	1.24 (1.18–1.30)

**Figure 2:**
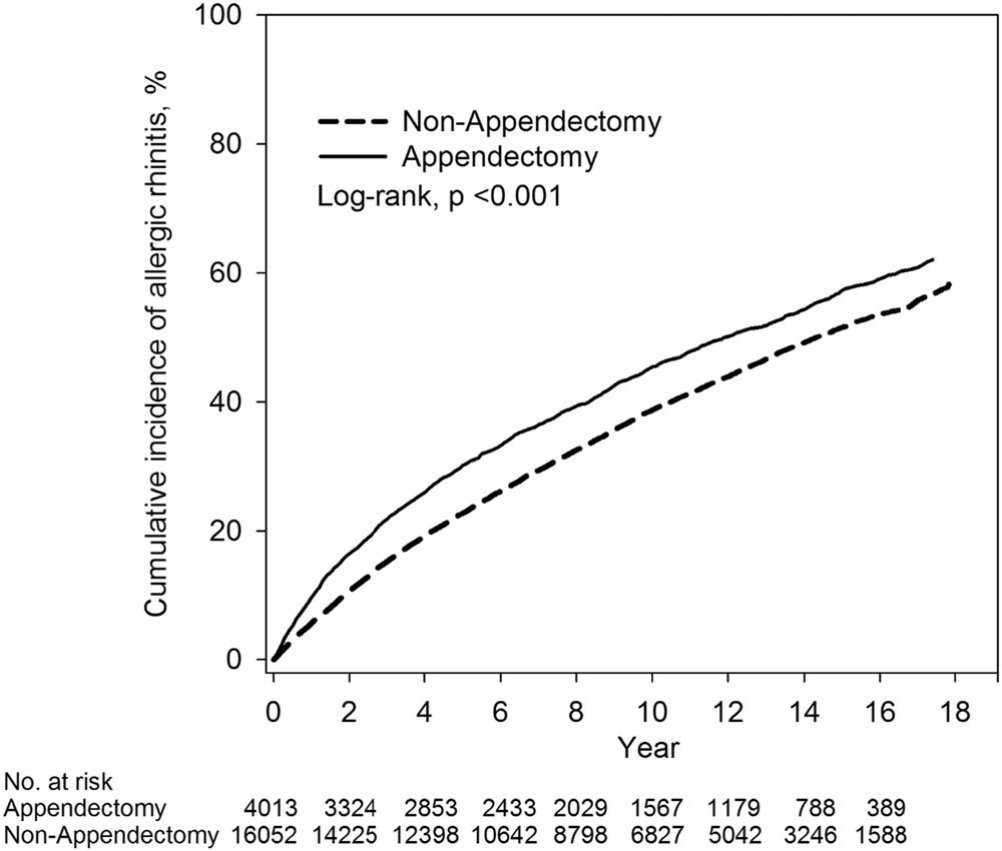
Cumulative incidence of allergic rhinitis between children with and without appendectomy.

In Figure 2, the Kaplan–Meier curves show the cumulative incidence of allergic rhinitis occurring after patients received appendectomy. The cumulative incidence for the development of allergic rhinitis was higher in the appendectomy group of the cohort than in the nonappendectomy group of patients. The *p* value of the log‐rank test was < 0.001 indicating a significant difference between the two groups.

We used Cox proportional hazard models analysis which included age, gender, area of residency, income, and various demographic characteristics in addition to appendectomy versus nonappendectomy, as shown in Table 3. After multivariable analysis, patients who underwent appendectomy had 1.23 times the risk of allergic rhinitis compared with the control group (HR = 1. 23; 95% CI, 1.17–1.29; *p* < 0.001), and females had a higher risk of allergic rhinitis than males (HR = 0.88; 95% CI, 0.84–0.92; *p* < 0.001). The risk of allergic rhinitis was higher at age 0–5 years compared to age 6–11 years (HR = 0.71; 95% CI, 0.64–0.78; *p* < 0.001) and age 12–18 years (HR = 0.59; 95% CI, 0.54–0.66; *p* < 0.001). Patients living in urban areas had a higher risk of allergic rhinitis than those living in suburban areas (HR = 0.96; 95% CI, 0.91–1.00; *p* = 0.04) and rural areas (HR = 0.95; 95% CI, 0.88–1.02, *p* = 0.14). The higher the income, the greater was the risk of developing allergic rhinitis (≧ NT $40,000, HR = 1.10; 95% CI, 1.04–1.17; *p* = 0.002; NT $20,001–$39,999, HR = 1.05; 95% CI, 1.00–1.10; *p* = 0.03). Moreover, patients diagnosed with ADHD (HR = 1.54; 95%CI, 1.15–2.06; *p* = 0.004), asthma (HR = 1.65; 95%CI, 1.20–2.25, *p* = 0.002), constipation (HR = 1.38; 95% CI, 1.05–1.81; *p* = 0.001) and intestinal (GI) Infectious disease (HR = 1.31; 95% CI, 1.06–1.61; *p* = 0.001), had a greater risk of developing allergic rhinitis.

**Table 3:** Cox proportional hazard model analysis for risk of allergic rhinitis.

	Univariable		Multivariable^†^	
	HR (95% CI)	*p* value	HR (95% CI)	*p* value
Group				
Nonappendectomy	Reference		Reference	
Appendectomy	1.23 (1.17–1.30)	< 0.001^†^	1.23 (1.17–1.29)	< 0.001^†^
Age (years)				
0–5	Reference		Reference	
6–11	0.70 (0.63–0.78)	< 0.001^†^	0.71 (0.64–0.78)	< 0.001^†^
12–18	0.59 (0.53–0.65)	< 0.001^†^	0.59 (0.54–0.66)	< 0.001^†^
Sex				
Female	Reference		Reference	
Male	0.89 (0.85–0.92)	< 0.001^†^	0.88 (0.84–0.92)	< 0.001^†^
Urbanization				
Urban	Reference		Reference	
Suburban	0.95 (0.91–1.00)	0.030^†^	0.96 (0.91–1.00)	0.04^†^
Rural	0.94 (0.87–1.00)	0.065	0.95 (0.88–1.02)	0.14
Monthly income (family)				
< NT $20,000	Reference		Reference	
NT $20,001–$39,999	1.05 (1.00–1.10)	0.052	1.05 (1.00–1.10)	0.03^†^
≥ NT $40,000	1.11 (1.04–1.18)	< 0.001^†^	1.10 (1.04–1.17)	0.002^†^
ADHD	1.49 (1.12–1.99)	0.006^†^	1.54 (1.15–2.06)	0.004^†^
Autism	0.89 (0.43–1.87)	0.766	0.69 (0.32–1.45)	0.32
Asthma	1.96 (1.44–2.67)	< 0.001^†^	1.65 (1.20–2.25)	0.002^†^
Atopic dermatitis	1.47 (0.99–2.20)	0.058	1.35 (0.90–2.01)	0.14
Diabetes	0.31 (0.04–2.20)	0.242	0.32 (0.05–2.26)	0.25
Chronic renal diseases	0.73 (0.33–1.63)	0.441	0.54 (0.24–1.20)	0.13
Chronic liver diseases	1.33 (0.84–2.11)	0.228	1.38 (0.87–2.19)	0.17
Constipation	1.53 (1.17–2.00)	< 0.001^†^	1.38 (1.05–1.81)	0.01^†^
Intestinal (GI) infectious diseases	1.47 (1.20–1.81)	< 0.001^†^	1.31 (1.06–1.61)	0.01^†^
Epilepsy	0.84 (0.55–1.30)	0.439	0.81 (0.53–1.24)	0.33

*Note:* ^†^Represent statistically significant.

The subgroup analysis is summarized in Table 4, which compares the incidence of allergic rhinitis between the appendectomy and nonappendectomy groups. The risk of allergic rhinitis after appendectomy was significantly higher in patients aged 6–11 years (HR = 1.20; 95% CI, 1.10–1.32; *p* < 0.001) and 12–18 years (HR = 1.25; 95% CI, 1.17–1.33; *p* < 0.001) in the exposed group compared with the control group. However, no statistically significant difference was reached in patients aged 0–5 years (HR = 1.14; 95% CI, 0.90–1.45; *p* = 0.28). Regardless of gender, the risk of allergic rhinitis was significantly higher in patients who had appendectomy (HR = 1.24; 95% CI, 1.15–1.34; *p* < 0.001 for women; HR = 1.23; 95% CI, 1.15–1.31; *p* < 0.001 for men). Among the areas of residency, the risk of allergic rhinitis after appendectomy was higher in the exposed group living in urban (HR = 1.25; 95% CI, 1.17–1.33; *p* < 0.001) and suburban areas (HR = 1.20; 95% CI, 1.10–1.31; *p* < 0.001). Compared with the control group, the risk of allergic rhinitis after appendectomy was higher in relatively lower income groups (< NT $20,000, HR = 1.16; 95% CI, 1.08–1.24, *p* < 0.001; NT $20,001–$39,999, HR = 1.40; 95% CI, 1.28–1.53, *p* < 0.001). When stratified by follow‐up duration, the risk of developing allergic rhinitis after appendectomy within 5 years of follow‐up was also significantly higher (< 1 year, HR = 1.71; 95% CI, 1.51–1.92; *p* < 0.001; 1–5 years, HR = 1.30; 95% CI, 1.20–1.40; *p* < 0.001).

**Table 4:** Subgroup for risk of allergic rhinitis.

	Nonappendectomy		Appendectomy			
	*N*	No. of allergic rhinitis	*N*	No. of allergic rhinitis	HR^†^ (95% CI)	*p* value
Age (years)						
0–5	488	327	122	86	1.14 (0.90–1.45)	0.28
6–11	4160	2128	1040	598	1.20 (1.10–1.32)	< 0.001^†^
12–18	11,404	4479	2851	1320	1.25 (1.17–1.33)	< 0.001^†^
					*p* for interaction = 0.567	
Sex						
Female	6680	3060	1670	896	1.24 (1.15–1.34)	< 0.001^†^
Male	9372	3874	2343	1108	1.23 (1.15–1.31)	< 0.001^†^
					*p* for interaction = 0.842	
Urbanization						
Urban	9012	3956	2198	1133	1.25 (1.17–1.33)	< 0.001^†^
Suburban	5419	2309	1350	651	1.20 (1.10–1.31)	< 0.001^†^
Rural	1621	669	465	220	1.23 (1.05–1.43)	0.009^†^
					*p* for interaction = 0.740	
Monthly income (family)						
< NT $20,000	8077	3723	2024	1030	1.16 (1.08–1.24)	< 0.001^†^
NT $20,001–$39,999	5537	2142	1367	670	1.40 (1.28–1.53)	< 0.001^†^
≥ NT $40,000	2438	1069	622	304	1.17 (1.03–1.33)	0.01^†^
					*p* for interaction = 0.002	
Follow‐up duration						
< 1 year	16,052	916	4013	386	1.71 (1.51–1.92)	< 0.001^†^
1–5 years	15,134	2657	3626	803	1.30 (1.20–1.40)	< 0.001^†^
> 5 years	11,473	3361	2617	815	1.04 (0.96–1.12)	0.32

*Note:* ^†^Represent statistically significant.

## 4 Discussion

In this large, population‐based cohort study, we noted that the relative risk of subsequent allergic rhinitis in patients who had undergone appendectomy was 1.24‐fold higher than in patients who had never undergone appendectomy.

The risk of allergic rhinitis was higher in individuals who were age 0–5 years, lived in urban areas or had a higher income. Patients diagnosed with ADHD, asthma, constipation, or intestinal (GI) infectious disease, also had higher risk of developing allergic rhinitis. The risk of allergic rhinitis was more significant in older patients after appendectomy, especially within 5 years of follow‐up.

Allergens can initiate an innate immune response by releasing alarmins such as interleukin (IL)−33, thymic stromal lymphopoietin (TSLP), or IL‐25. Type 2 cytokines (IL‐5, IL‐13, and IL‐4) are produced by activating group 2 innate lymphocytes (ILC2s) in response to alarmins. In addition, allergen‐specific TH2 cells activate B cells, IgE class switching, and mucosal inflammation which play the most important roles in allergic rhinitis [12, 13, 16, 17, 22].

As Bousquet et al. [17] and Arasi et al. [19] mentioned in their review articles, allergic rhinitis is more common in high‐income countries, and is relatively less prevalent in low‐ and middle‐income countries. This observation is compatible with our study, which showed that the risk of allergic rhinitis was higher in patients living in urban areas with a higher income. This may be explained by the greater air pollution and higher amount of allergens in the towns and cities than in rural areas.

There are two possible mechanisms that could explain the association between appendectomy and the risk of allergic rhinitis: alteration of immune responses and gut microbiota dysbiosis. According to the “common mucosal immune system” hypothesis proposed by Bienenstock [23], the mucosal immune system throughout the human body is an interconnected system. In this system, immune cells from different mucosal tissues could be regarded as the same organ system. Human immune cells promote effective interaction and cooperation between various mucosal sites to defend against external stimuli and maintain the host's immune stability. These mucosal tissues include those of the gastrointestinal tract, respiratory tract, and urogenital system. Several studies have demonstrated that the microbiota has an impact on allergic diseases because it plays an important role in modulating immunity and inflammatory responses. Hence, gastrointestinal dysbiosis is significant in patients with allergic rhinitis and the intestinal microbial diversity is also significantly reduced [16, 24, 25, 26]. Gastrointestinal dysbiosis and decrease in intestinal metabolites (such as short‐chain fatty acids (SCFAs) and tryptophan) induce the production of TSLP, IL‐25, and IL‐33, thereby promoting the production of Th2 cytokines and local inflammatory responses [16]. Some strains of microbiota inhibit the proliferation and invasion of pathogenic microorganisms by releasing antimicrobial bacteriocins and other species produce SCFAs which induce regular T cell (Treg) to balance pro‐inflammatory and anti‐inflammatory pathways [10]. Thus, in recent years, probiotics have been suggested as immunomodulators to alleviate allergic reactions by influencing phagocytosis and the production of pro‐inflammatory cytokines, thereby preventing allergic diseases [11, 12, 13, 14, 16].

In recent years, growing evidence indicates that the human appendix plays an important biological role in regulating the immune system and biofilm of the intestine and it is therefore no longer regarded as a nonfunctional vestigial organ [2, 3]. Several species of microorganisms lived in the appendix including *Eubacterium rectale*, *Akkermansia muciniphila*, Firmicutes, Bacteroidetes, Actinobacteria, and Proteobacteria species [2, 7]. Appendectomy may cause changes in bacterial and fungal species within the intestinal flora and affect interactions between bacteria and fungi. This kind of dysbiosis of the microbiota in the intestine alters intestinal metabolic activity and vulnerability to pathogens which can result in gastrointestinal tract cancer, inflammatory bowel disease, and other systemic diseases, including obesity, allergic disease, diabetes, sepsis, and even psychiatric disorders [2, 4, 5, 6]. Indeed, dysregulation of the microbiota of the gastrointestinal tract has a significant negative impact on the host immune system. The typical gut microbiota in allergic rhinitis patients alters cytokines, which leads to Th2 predominance [9, 12].

In addition, the appendix is a gut‐associated lymphoid organ (GALT) which contains a large amount of lymphoid tissue including T and B lymphocytes according to studies conducted in the early 2000s [2, 3, 9, 27]. This lymphatic development begins from 2 weeks after birth by stimulation of antigen exposure and atrophies after 30 years old. Immunoglobulin (Ig) A is mainly produced in the appendix of the GALT system. The majority of IgA is in the gastrointestinal tract which eliminates harmful bacteria and viruses by binding to pathogens and toxins strongly and facilitates their elimination. It also plays an important role in the prevention of allergens transferring into the bloodstream directly. Thus, when the appendix is removed during the neonatal period, the growth of the lymphatic tissue of the immune system is affected. In a study by Andreu‐Ballester and colleagues, a significant reduction in secretory IgA levels in serum was found after patients received appendectomy, and it may persist for 3 years or more [28]. Also, in selective IgA‐deficient patients, secretory IgM and IgE are increased to compensate for the IgA deficiency [29]. Several studies have stated that these patients may develop recurrent upper respiratory infections, autoimmune disease, and allergies [3, 11, 29, 30, 31, 32].

Although Table 3 shows that the incidence of allergic rhinitis diagnosed in children aged 7–11 and 12–18 is lower than that in children aged 0–6, which is different from previous literature, it may be related to the children seeking medical treatment in Taiwan and the use of ICD‐9‐CM code to define allergic rhinitis. There might be an underestimation in these two older groups.

According to the results of the stratified analysis in Table 4, the older the age, the greater the chance of undergoing appendectomy. The reason might be that acute appendicitis is more common in older children and adolescents [21]. Patients who undergo surgery may need to go to the hospital for follow‐up for a period after the operation. The increased frequency of medical visits may lead to a higher diagnosis rate of allergic rhinitis (AR) might be increased due to the increased medical treatment. Therefore, the prevalence is positively correlated with age, which is consistent with previous literature [33, 34, 35]. This trend is believed to be attributed to complex mechanisms, primarily centered on the development of the pediatric immune system. As we age, we are exposed to more and more types of environmental allergens and for longer periods. The early‐life microbial exposure influences immune maturation, potentially skewing toward Th2‐mediated allergic responses [34, 35]. Besides, increasing cumulative exposure time to some allergens (e.g., dust mites, pollen, mold) naturally increased the likelihood of sensitization [36].

Since the appendix still plays an important role in the immune system and gut microbiome, the timing of appendectomy for acute appendicitis is critically important. Traditionally, we rely on patients' chief complaints, physical examination, and imaging examination (such as abdominal ultrasound or CT) to diagnose acute appendicitis. Recent articles have proposed that some inflammation markers may reflect immune and inflammatory status and may be not only helpful to diagnosis acute appendicitis more precise but also predict the severity of acute appendicitis [37, 38]. Siki et al. [39] suggested that the systemic immune inflammation response index (SIRI) and systemic immune inflammation index (SII) may be helpful to diagnose acute appendicitis more accurately. The SIRI means the (neutrophil count × platelet count)/lymphocyte count and the SIRI is the neutrophil count × monocyte count/lymphocyte ratio. Higher index scores may indicate the need for early surgical intervention, thereby reducing false‐positive diagnoses. Incorporating more medial information ensures the correct diagnosis of acute appendicitis and prompt surgical intervention, which may avoid complications caused by unnecessary appendectomy [40]. The major strength of this study was the large size of the cohort and the availability of a complete history of medical services especially surgical intervention from the NHIRD which is a huge electronic healthcare database in Taiwan. Patients received a relatively long‐term follow‐up duration. In addition, confounding variables including selection, recall, and participation bias were adjusted by rigorous exclusion criteria and 1:4 propensity score matching. Finally, the detailed subgroup analysis also supported the finding that there was a high risk of allergic rhinitis in patients who had previously received appendectomy.

Nevertheless, there were still some limitations in this study. First, the patients' lifestyle and habits, such as dietary habits or use of health supplements such as probiotics, are not recorded in the NHIRD, and these may be risk factors affecting the development of allergic rhinitis. Unmeasured confounding bias may still have existed, despite adjusting for various comorbidities, socioeconomics, urbanization of living place, and matched propensity scores. Second, the diagnosis of allergic rhinitis relied solely on ICD‐9 code, as determined by physicians, and thus it was not possible to confirm the accuracy of diagnosis. As it is not possible to verify the accuracy of diagnosis by reviewing medical records in the NHIRD, there may have been an overestimation of the prevalence of disease. Indeed, if patients only had mild symptoms of allergic rhinitis, they may not have sought medical services potentially resulting in an underestimation of the disease. Third, the NHIRD is Taiwan's exclusive medical record database, and the results of this study may not be generalizable to other countries or populations with different ethnicities.

In conclusion, patients who underwent appendectomy were 1.24 times risk more likely to develop allergic rhinitis than the control group without appendectomy, especially within 5 years of postoperative follow‐up. This finding was supported by a detailed subgroup analysis. In addition to appendectomy, we found that age younger than 5 years old, female, living in an urban area, and comorbidities including asthma and gastrointestinal diseases, may affect the development of allergic rhinitis. Therefore, we suggest that the indication of appendectomy should be carefully evaluated to decide the best way to treat acute appendicitis. Patients should be closely monitored for symptoms of allergic rhinitis after appendectomy, and medication treatment should be given as soon as possible.

## Author Contributions

**Wen‐Chun Lin:** conceptualization, methodology, writing – original draft, writing – review and editing. **Meng‐Che Wu:** conceptualization, methodology, validation. **Yu‐Hsun Wang:** data curation, formal analysis. **James Cheng‐Chung Wei:** supervision, visualization. All authors have read and approved the final version of the manuscript.

## Ethics Statement

The Research Ethics Committee at Chung Shan Medical University and Hospital in Taiwan approved the study (CSMUH No: CS1‐20201). The study followed the principles of the Declaration of Helsinki.

## Consent

The consent written from study subjects was proved to be waived by the Institutional Review Board of the Research Ethics Committee of Chung Shan Medical University and Hospital.

## Conflicts of Interest

The authors declare no conflicts of interest.

## Transparency Statement

The lead author Wen‐Chun Lin affirms that this manuscript is an honest, accurate, and transparent account of the study being reported; that no important aspects of the study have been omitted; and that any discrepancies from the study as planned (and, if relevant, registered) have been explained.

## Data Availability

The database used in this study is held by the Taiwan Ministry of Health and Welfare (MOHW). The MOHW must approve our application to access the data, and patient consent was exempted as a result of de‐identification of the NHIRD (Database NHIR, Taiwan, available at http://nhird.nhri.org.tw/en/index.htm). Any researcher interested in accessing this data set can apply form to the MOHW requesting access. All relevant data are within the paper. Wen‐Chun Lin had full access to all of the data in this study and takes complete responsibility for the integrity of the data and the accuracy of the data analysis.
